# Carcass Color in Broilers When Replacing Wheat with Corn in the Diet

**DOI:** 10.3390/foods14152558

**Published:** 2025-07-22

**Authors:** Maria del Mar Campo, Leticia Mur

**Affiliations:** Department of Animal Production and Food Science, Instituto Agroalimentario de Aragón-IA2, Universidad de Zaragoza-CITA, C/. Miguel Servet 177, 50013 Zaragoza, Spain; leticiamurpalus@hotmail.com

**Keywords:** broiler, color, carcass, corn, wheat

## Abstract

The effect of replacing wheat with corn on the color distribution across various locations in the carcasses of broilers was assessed. One thousand two hundred ROSS 308 one-day-old male chicks were reared in an experimental farm, with ten pens per treatment, based on the primary cereal ingredient during both the starter (1–14 d) and grower (15–41 d) phases: corn and wheat. At 41 days old, slaughtering was performed. At 24 h post slaughter, color measurements were taken in 15 carcasses per treatment using a MINOLTA CM600d reflectance spectrocolorimeter in the CIEL*a*b* space. Twelve locations were measured: drumette, apterial latero-pectoral area, neck, the back at the seventh thoracic vertebra, saddle, thigh, ham, hock and around the vent, shanks, and the surface and interior of the breast. The results indicate that the color distribution in the broiler carcass is not homogeneous. Very small color differences can be found between the surface and the interior of the breast, but they differ greatly from areas where there is skin. Corn produces a darker color than wheat, though the intact skin homogenizes lightness* across the entire carcass, reducing differences between regions. Corn increased mainly yellowness, influencing color saturation. The pronounced color differences between the shank and other locations almost disappear when corn is excluded from the feed. Hue° could serve as a potential indicator of color variations in the breast resulting from differences in dietary ingredients.

## 1. Introduction

Appearance is one of the most important quality attributes that drives consumer behavior at purchase and, in most cases, is the only characteristic that can be assessed when meat is displayed in sealed packaging at the supermarket. Appearance encompasses both physical aspects and color, and both are crucial for the consumer when judging freshness [1]. This judgment is mainly associated with the characteristic pink color of the chicken meat [1], but this color only applies to what is referred to as white chicken. Some consumers have traditionally looked for white-skinned chicken, while others are used to consuming yellow-skinned chicken, even within the same country [2,3]. In Spain, in the early 1980s, most of the chicken in the market was yellow. Nevertheless, from 2000 onwards, only some areas in the north of the country maintained this product at retail as a remembrance of traditional husbandry practices, whereas in the rest of the country, most consumers could only find white-skinned chicken at retail. Currently, the trend is slowly shifting toward a higher presence of yellow-skinned chicken in supermarkets, with increased added value associated with a natural product. This shift in production is growing due to a different type of consumer, more focused on animal welfare [4], even if they have limited knowledge of how these animals are produced [5]. Additionally, some consumers associate a yellow color with a healthier animal [6], as only healthy animals absorb pigments that can be transported in the blood to skin and fat tissues [7]. These skin colors depend on the animal’s diet, linked to the presence of carotenoids that cannot be synthesized by the animal [8], as well as the animal’s capacity to absorb and deposit them [9]. These compounds form the largest group of naturally occurring pigments, and are responsible for yellow to red colors in fruits, vegetables, algae, or animals. Some of them also exhibit antioxidant activity or vitamin A activity once metabolized through conversion from monohydroxy- or monoketocarotenoids [10].

Corn is a common ingredient in poultry diets. Carotenoids present in corn include lutein, zeaxanthin, *β*-cryptoxanthin, *β*-carotene, and *α*-carotene [11], which are responsible for its hue. In chicken, *β*-carotene is metabolized into vitamin A; therefore, it contributes little to meat pigmentation [10]. For yellow-skinned chicken, consumers have become accustomed to a very intense yellow skin that may even turn orange at times [12]. To achieve this high-intensity color in chicken, the industry combines natural and/or synthetic yellow and red carotenoids [2,7] in a diet that also includes corn as an ingredient. This ensures that the color does not appear artificial and helps to reduce the price of the ration if the inclusion rate is not too high. This approach is particularly relevant within the European Union, where the feed is based on wheat, in contrast to the USA, where the feed is based on corn due to higher availability. Previous researchers have conducted studies on the effect of varying levels of natural or synthetic carotenoids in the diet on the performance or quality of the meat [7,13,14,15]. Nevertheless, the effect that corn has on the color of the final product has faded out, or even been forgotten, due to the supplemented carotenoids in the feed [16]. Consumers are not aware of the mechanisms that lead to the desired color, believing that it is solely due to the intake of corn. However, a movement among consumers toward natural food production and reducing unnecessary additives in the feed could eliminate the inclusion of extra carotenoids in the formulation. This would also reduce costs as additives, particularly synthesized ones, can be expensive. Corn and wheat prices fluctuate throughout the year depending on availability. In Spain, there is a high dependence on foreign sources for both ingredients, and geopolitical events—such as those occurring in Ukraine—can influence the cost of these commodities. At the time of this project, the prices of wheat and corn in Spanish raw material markets were EUR 196 per ton and EUR 178 per ton, respectively. Therefore, this study was conducted with the aim of assessing the effect of the substitution of wheat by corn in the diet of modern broilers on the color of different locations in the carcass, without supplementing additional carotenoids, with the hypothesis of a heterogeneous distribution of color across the carcass, using the CIEL*a*b* system as a non-invasive methodology.

## 2. Materials and Methods

### 2.1. Animals

The Ethics Committee for Animal Experiments at the University of Zaragoza approved this project (PD19/20NE). Animals were reared in the Experimental Unit of UVESA in Tudela (Navarra), divided into 20 pens (2 × 2.5 m), with 60 ROSS 308 1-day-old male chicks per pen. Animals were randomly distributed in the pens on the day of arrival. Pens were alternatively assigned to one of two diets characterized by their main cereal ingredient in both phases, corn and wheat, for a total of ten pens per treatment. Breeding conditions were those commonly used in the industry. From the first to the fourth day, animals were fed on papers on the floor that were simultaneously set up with the feeders. Throughout the trial, broilers had access to feed and water ad libitum. Animals received two different standard broiler diets per treatment: starter from 1 to 14 days old, and grower from then onwards until reaching 41 days old. Diets were formulated to be isoenergetic and isonitrogenous based on digestible lysine. The ingredient and nutrient composition of the diets can be found in Table 1. No digestibility problems were observed with these levels of wheat due to the addition of carbohydrases. Animals were exposed to 24 h of light above 60 lx the first three days, with 2 h of darkness added starting on day 4, reaching a total of 6 h of darkness by day 7. The temperature decreased from 33 °C at the beginning of the trial to 20 °C at its conclusion.

When the animals reached 41 days old, they were transported for less than 30 min to the abattoir after fasting on the farm for 6 h, with total feed withdrawal for less than 10 h, including lairage. Stunning was performed with CO_2_. Carcasses were defeathered, eviscerated, and had their heads, shanks, and vents removed prior to chilling. Shanks were randomly collected and chilled at this point.

### 2.2. Carcass and Color Analyses

**Figure 1:**
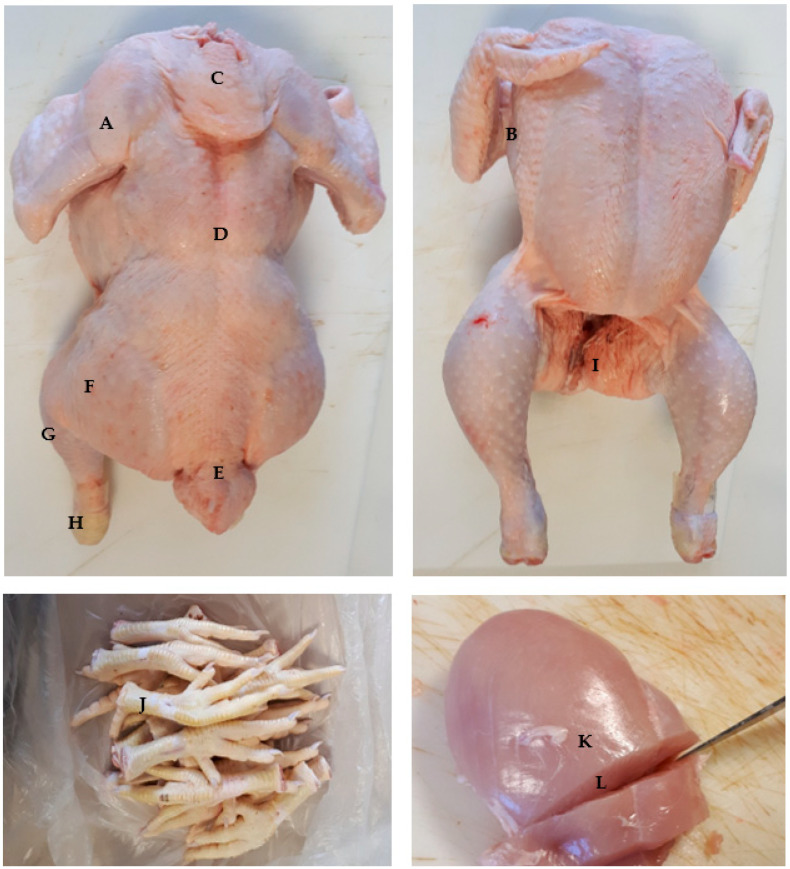
Regional locations of measurements: (**A**) drumette; (**B**) apterial latero-pectoral area; (**C**) neck; (**D**) back; (**E**) saddle; (**F**) thigh; (**G**) ham; (**H**) hock; (**I**) vent; (**J**) shank; (**K**) breast surface; (**L**) breast interior.

At 24 h after slaughter, 15 carcasses per treatment, randomly chosen from the pens, were transported under commercial refrigerated conditions at 4 °C from the processing plant of the abattoir to the facilities of the meat quality laboratory at the Veterinary Faculty of the University of Zaragoza. Carcasses were weighed. Color was measured at 12 points: 9 different points on each carcass: drumette, apterial latero-pectoral area, neck, the back at the 7th thoracic vertebra, saddle, thigh, ham, hock and around the vent, as well as on the shanks, which were automatically separated from the carcass at the abattoir, and on the surface and interior of the breast after dissection from the carcass (Figure 1). The reflectance spectrocolorimeter MINOLTA CM600d (Konika Minolta, Inc., Tokyo, Japan) in the CIEL*a*b* color space [17] with illuminant D65 and a 10° standard observer was used, obtaining the average of three measurements in each location per carcass. Lightness (L*), redness (a*), and yellowness (b*) were recorded, and Chroma [C*, √(a^2^ + b^2^)] and Hue angle (H°, tan^−1^(b*/a*) in degrees) were calculated. The color difference, ΔE, which reflects the total color change between the dietary groups, was also calculated with the equation

ΔE = √(ΔL*^2^ + Δa*^2^ + Δb*^2^)

where ΔL* = L*Wheat—L*Corn; Δa* = a*Wheat—a*Corn, and Δb* = b*Wheat—b*Corn.

The breast was weighed, and the carcass breast yield was calculated. pH was then measured with a penetrating electrode Labprocess PC7 XS. Finally, the degrees of wooden breast (WB), white striping (WS), and spaghetti meat (SM) breast myopathies were assessed on a 3-point scale: 0, absence; 1, moderate; 2, severe [18].

### 2.3. Statistical Analyses

Using SPSS 26.0, a General Linear Model (GLM) was applied to assess the effect of diet and region on carcass, pH, and color parameters. Differences within mean values were analyzed with a Tukey’s multiple range test. Kruskal–Wallis was used to assess differences in breast myopathies. A Principal Component Analysis was also used to study variability and the relationships amongst the variables at each diet.

## 3. Results and Discussion

No significant differences were found in carcass weight or breast yield (Table 2) between treatments. pH was not different between diets. A pH over 6.1 would have been considered high, proper for DFD meat, whereas a pH below 5.7 would have been considered low and characteristic of PSE meat. Both abnormal pHs would have influenced the color, making it darker or lighter, respectively. Nor was the presence of the three studied breast myopathies significantly different between treatments (Table 2). Wooden breast and white striping have been described to increase L*, a*, and b* values [19], due to edema as a consequence of the inflammatory process [20], changes in the structure of the fiber [19], and the lipid accumulation that increases the depots of liposoluble pigments from the feed [19], among other factors. Therefore, we can assume that differences found in color were due to the dietary treatments analyzed.

The interaction between the regional location and the dietary treatment was highly significant (*p* < 0.001) for a*, b*, Ch*, and H^o^, and significant for L* (*p* < 0.05). Therefore, all data are shown in relation to dietary treatment and regional location.

Lightness was affected by the diet in five out of the twelve locations analyzed (Table 3). In all of the significantly different regions in color, wheat produced a lighter color than a corn-based diet, especially in the neck, the back, and the shank (*p* < 0.001), but also in the drumette and the apterial latero-pectoral area (*p* < 0.05). In all these areas, differences between diets exceeded 1.4 points. Consumers will differentiate between diets due to darker and lighter carcasses in these locations because the human eye is able to distinguish lightness differences greater than one point when colors are compared next to each other, but they might not be able to differentiate the breast, which is the most expensive piece, either whole or sliced. Neither Rajput et al. [15] nor Peng et al. [21] found differences in lightness in the meat of the breast or the thigh with increasing levels of marigold supplementation or with different grain sources, respectively. On the contrary, Lyon et al. [22] also obtained significantly lighter breasts in animals fed wheat than corn or sorghum, but in this case, in thawed meat, they obtained a darker color than in our study, which could reflect the influence of freezing and thawing on color development.

The darkest location was the breast, either inside or on the surface, irrespective of the diet, followed by the ham. All the other locations showed lightness* higher than 71 in corn or 72 in wheat diets, with the shank in wheat and the hock in corn diets being lighter than the neck. These shank L* values are in the same range as those found by Peng et al. [21] in corn- and sorghum + barley-based diets. But these authors found values above 71 in the breast skin, whereas we found L* values below 56 in the breast muscle, coincidentally with Ciurescu et al. [23]. In our study, we did not measure the skin when measuring the breast (Figure 1), considering that most consumers at retail purchase skinless breasts. In fact, except for the ham, which has very thin skin, all the other locations showed high lightness values that are comparable to those found by Peng et al. [21]. Therefore, with the exception of the leg, whole carcasses can be considered fairly homogeneous in lightness*, irrespective of the diet, when considering the color over the intact skin.

Significant differences appeared in the a*index between diets in all regions (Table 3), except in the saddle (*p* = 0.137) and the thigh (*p* = 0.062). As expected, corn provoked higher redness than wheat, whose lower a* values could even be negative. This relates to the carotenoid content of the grains, with extremely low content in wheat and very high content in corn (12 mg/kg in the corn of our study), especially when fortified with carotenoids [24]. In fact, provitamin A carotenoids (α- and β-carotene and β-cryptoxanthin) or zeaxanthin are not detected in traditional wheat varieties [25]. Nevertheless, the carcass is not homogeneous in redness. Values in the breast were almost 3-fold lower than those found by Córdova-Novoa et al. [26], perhaps because their animals were 10 days older, and pigmentation increases with age or is reduced when restricted in the feed [13].

The breast and the posterior part of the body (ham, shank, thigh, and hock) showed lower redness than the saddle and around the vent, due to the higher fatness in those latter areas. The dynamics of adipose tissue in different areas of the body have been found to be age-related [27], with differences in growth rate after hatching, both by hyperplasia and hypertrophy of adipocytes. Abdominal fat is a late-developing adipose tissue in comparison with clavicular and subcutaneous fat [27]. Therefore, pigments in the diet will accumulate to a greater extent in late-developing tissues when administered in the grower feed, as was the case with the incorporation of corn as the main ingredient in this phase, which coincided with the latest feed administered to the animal.

The largest differences between diets appeared in yellowness (Table 3) in all locations except the thigh (*p* = 0.237) and the ham (*p* = 0.320), with the latter location showing the lowest yellowness intensity in the corn diet. Lutein and zeaxanthin (yellow pigments with absorbance at 445–450 nm) are the major carotenoids in corn [28], contributing to a larger extent to the development of yellow hue than of red hue. The shank in the corn group showed a 60% higher b* value than in the wheat group, and it was the region with the highest b* value [31.6 vs. 18.7, respectively]. Some authors have not found differences in any chromatic values in the breast [26] or in the breast and the thigh [23] when substituting corn with other cereals, such as sorghum. Although the total carotenoid content in sorghum is significantly lower than in yellow corn, its bioavailability is higher [29]. The fact that lutein and zeaxanthin are the major carotenoids in sorghum [29], as in corn, might contribute to similar L*a*b* values when comparing both cereals in the diet.

**Figure 2:**
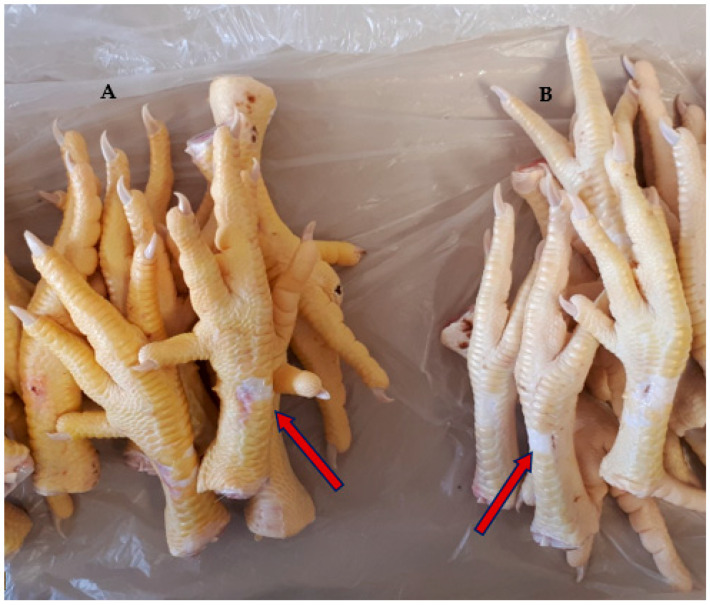
Shanks from the corn (**A**) and wheat (**B**) groups, with remarks on areas that lost scales during processing and were avoided in the measurements.

A higher amount of yellow pigments in the diet increased the differences between the shank and the subsequent locations, such as the vent, which were very large (8.56 points) in the corn group but very small (0.05 points) and not significant in animals from the wheat group. This might be due to the fact that zeaxanthin and canthaxanthin are mainly deposited in the shanks [30]. When zeaxanthin is supplemented in the diet, yellowness in the shank can rise by over 35 units, reaching even 65 when administered together with canthaxanthin [7]. Yellowness can reach 44 units in the hock [31] or in the breast [12] when marigold is supplemented in the feed, far higher than the values of just over 10 found in our study in the corn diet without further carotenoid supplementation. The scales in the shank are structures that are able to incorporate carotenoids very early in animal development. However, in the abattoir, during the defeathering process at the shackle line, they can be removed due to physical contact with the rotating rubber fingers used for plucking the feathers, resulting in an irregular, whiter appearance of the shank (Figure 2), thus diminishing visual differences between the treatments. Nevertheless, this can be considered an undesirable carcass quality defect and was avoided in our measurements.

Chroma* reflects the saturation or concentration of a specific color. As a result of the differences in redness and yellowness, large differences appeared in Chroma* between diets (Table 4), except in the thigh and the ham, which showed small differences (1.37 and 0.74 units, respectively, in each location between the wheat and corn groups). Again, the shank is the location with the largest differences between diets (13.1 units), followed by the hock (5.27 units) and the neck (5.06 units). The intensity of the saturation will be linked to the deposition rate of those carotenoids and pigmentation properties in the tissues. Some competition among carotenoids has been described for uptake, absorption, and incorporation into micelles [32], favored by dietary fat. However, Hencken [10] found that most carotenoids are excreted without absorption, and only 1.7% of zeaxanthin is deposited in the skin. This may happen, among other factors, due to the saturation of the uptake with high carotenoid doses or the physiological turnover of mucosal cells, the maturity of the enterocytes, or the morphology of the mucosa [32]. If there is a microbial disorder, feed utilization will be reduced [33]. Some organs, such as the retina or liver, will contain more carotenoids than adipose tissue, either at high or low intake doses [34,35]. Therefore, it seems logical that the small differences found between diets in those locations with thin skin or low subcutaneous fat would occur, since the difference in deposition of certain carotenoids will be minimal between the diets with high and low content, after tissue competition for the deposition of the pigments.

In addition to the shank, higher Chroma* was found around the vent, the hock, the saddle, and the neck, with low values in the ham and the breast. Few authors calculate this saturation value in their work, which is highly dependent on b* intensity due to its predominance over the a* value in pigmented chicken. In a sorghum diet, Ponsano et al. [14] found comparable saturation in the thigh and the breast to those found in the corn group. Calculated data reflect similar values in corn or sorghum diets in the breast and the thigh [23], but much lower color saturation in the thawed breast [22] between corn- (4.16), sorghum- (2.95) and wheat- (2.57) based diets than those found in our work, indicating color losses after the freezing process.

Hue° reflects the color perception by which an object is red, blue, and so on [14]. As with Choma*, few authors calculate this chromatic attribute, but it is an important discriminator of the color of the meat with abnormal pH, as an example [36]. Although differences were obvious between diets in L*a*b* and Chroma* values, Hue° did not differ in the drumette, apterial latero-pectoral area, around the vent, the saddle, or the thigh (Table 4) between the corn and wheat groups. The largest differences appeared in the breast and, especially, the ham, with higher values in the wheat group in every location. The ham had the highest Hue° in the wheat group, whereas the thigh had the highest in the corn group, although it was no different from the interior of the breast in both groups and the ham in the case of the corn group. After calculating this attribute from published data, these differences in Hue° have also been found in the breast between diets when comparing corn with sorghum and wheat [22,23,26], which could be an indicator of different feed sources while assessing color differences in the carcass.

**Figure 3:**
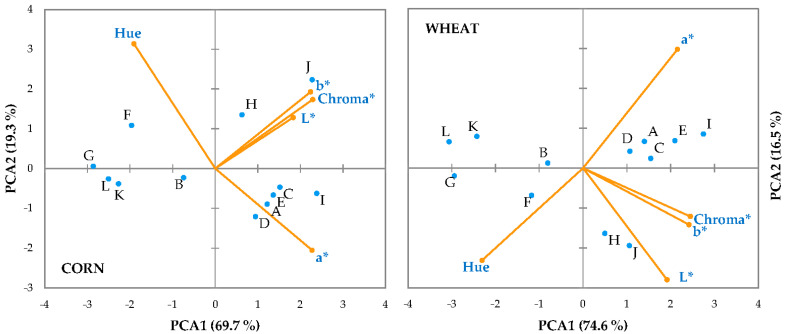
Principal Component Analysis loading plots in the planes of components 1 and 3 for color parameters in the corn and wheat groups at different regional locations: (**A**) drumette; (**B**) apterial latero-pectoral area; (**C**) neck; (**D**) back; (**E**) saddle; (**F**) thigh; (**G**) ham; (**H**) hock; (**I**) vent; (**J**) shank; (**K**) breast surface; (**L**) breast interior.

The Principal Component Analysis explained 91.1% of the color variability in the wheat group and 88.0% in the corn group within the first two axes (Figure 3). The association of color variables was similar regardless of diet, with Chroma* clearly associated with the b* index and L*, while the a*index was inversely related to Hue°. However, the distribution along PCA2 was opposite in both groups, with the a* index positioned positively in the wheat group and negatively in the corn group. This corresponded to the location of different regions, which were inversely positioned depending on the diet according to PCA2.

Those areas with higher color intensity, such as the shank and the hock, were closely related to Chroma*, b*, and L* parameters. These regions were positively associated with PCA2 in the corn group and negatively associated with PCA2 in the wheat group. They were inversely related to the apterial latero-pectoral area and, especially, the breast, indicating very different colors in these locations. The regions of the hind limb were separated from the other regions by PCA2, with the thigh and ham grouped together but separated from the hock and the shank by PCA1.

These differences can be also observed with the ΔE parameter (Table 4), which helps to understand how the human eye perceives color differences. The ham and the thigh showed the lowest color differences between diets (1.12 and 2.00, respectively). Considering that a ΔE value less than 1 is generally imperceptible by the human eye, and values between 1 and 2 are perceptible only upon close observation, we can consider that the differences in a* values at these two locations between dietary treatments are not visually significant. The saddle also exhibited a small ΔE value. Therefore, these three locations on the hind part of the body have very similar colors.

All other locations had ΔE values exceeding 3.0, which is high enough to be noticeable to the average person [37]. The largest difference was observed in the shank (13,75), as clearly shown in Figure 2.

## 4. Conclusions

As a conclusion, color distribution in the carcass in the broiler is not homogeneous. Very small color differences can be found between the surface and the interior of the breast, but they differ greatly from locations where the skin is valued. Corn produces a darker color than wheat, although the intact skin homogenizes lightness* across the entire carcass. Pigments from the corn present in the feed without further carotenoid supplementation mainly increase yellowness, influencing color saturation. The large differences between the shank and the rest of the locations due to carotenoids almost disappear when corn is not included in the feed and, therefore, low pigments are consumed. Hue° could be an interesting indicator of differences in the color of the breast due to variations in the ingredients of the feed. Further research can optimize the type of ingredients in the feed by assessing the influence of various raw materials with different carotenoid contents across different broiler strains to achieve consumer acceptability, thereby reducing the need for the inclusion of pigments as additives in the diet.

## Figures and Tables

**Table 1:** Ingredients and nutrient composition of experimental diets (g/kg as fed basis).

	Starter (0–14 d) *Crumble*		Grower (15–41 d) *Pellet*	
	WHEAT	CORN	WHEAT	CORN
*Ingredients %*				
Corn	5.00	30.0	0	45.0
Wheat	30.0	15.0	45.1	0
Barley	20.0	6.70	25.0	22.9
Soybean meal, 47%	30.0	30.0	20.8	25.4
Full-fat soybean meal	5.00	6.00	0	0
Soybean oil	0	0	2.51	2.25
Sunflower meal, 34%	3.00	4.00	3.00	1.00
Tallow	1.60	1.60	0	0
Mucose, hydrolyzed	2.00	2.65	0	0
Dicalcium phosphate	0.920	0.968	0.965	1.10
Limestone	0.786	0.712	0.981	0.871
Vitamin–mineral premix ^1^	0.500	0.500	0.500	0.500
MHA-Ca ^2^	0.447	0.447	0.327	0.346
L-Lysine HCl	0.278	0.260	0.293	0.233
Salt (NaCl)	0.168	0.153	0.167	0.217
Sodium bicarbonate	0.119	0.100	0.189	0.137
L-Threonine	0.130	0.115	0.106	0.071
*Calculated nutrients %*				
Dry matter	89.5	88.4	90.0	87.9
ME poultry, kcal/kg	2980	2980	3064	3050
Crude protein	29.0	23.8	18.7	18.3
Lysine, total	1.47	1.47	1.09	1.10
Lysine, digestible	1.31	1.31	0.970	0.970
Crude fiber	3.91	3.81	3.72	3.15
Crude fat	4.09	4.82	4.00	4.77
Ash	5.54	5.51	5.10	5.00
Calcium	0.965	0.950	1.00	0.995
Phosphorus, total	0.597	0.609	0.551	0.553
Phosphorus, digestible	0.440	0.440	0.420	0.420

^1^ Contains per kg premix: vitamin A, 2,000,000 IU; vitamin D3, 700,000 IU; vitamin E, 10,000 IU; vitamin K3, 0.6 g; thiamine, 0.5 g; vitamin B2, 1.4 g; vitamin B6, 0.8 g; vitamin B12, 4 mg; biotin, 0.1 g; folic acid, 0.3 g; niacin, 16 g; d-pantothenic acid, 2.8 g; choline chloride, 50 g; betaine anhydrous, 24 g; Fe (iron sulfate monohydrate), 8 g; Cu (copper sulfate pentahydrate), 3 g; Zn (zinc sulfate monohydrate), 12 g; Zn (zinc sulfate monohydrate), 8 g; Mn (manganese sulfate monohydrate), 20 g; Se (sodium selenite) 0.06 g; I, 0.2 g; endo-1,4-beta xylanase, 390,000 EPU; 6-phytase, 300,000 FTY; narasin, 10 g; nicarbazin, 10 g. ^2^ Methionine hydroxy analogue–calcium salts.

**Table 2:** Carcass characteristics, pH, and myopathies (number of observations) in broilers fed wheat- or corn-based diets.

	FEED		SEM	*p*
	WHEAT	CORN		
*n*	*15*	*15*		
Carcass weight (kg)	2.15	2.27	0.306	0.279
Carcass breast yield %	33.2	32.8	2.05	0.573
pH	5.89	5.90	0.107	0.842
Wooden breast				
Normal	10	12		0.378
Moderate	1	2		
Severe	4	1		
White striping				
Normal	14	12		0.291
Moderate	1	3		
Severe	0	0		
Spaghetti meat				
Normal	13	15		0.150
Moderate	1	0		
Severe	1	0		

**Table 3:** Lightness (L*), redness (a*), and yellowness (b*) of different carcass areas in broilers fed wheat- or corn-based diets.

	Lightness		SEM	*p*-Value	Redness		SEM	*p*-Value	Yellowness		SEM	*p*-Value
	WHEAT	CORN			WHEAT	CORN			WHEAT	CORN		
*n*	15	15			15	15			15	15		
Apterial latero-pectoral area	74.43 ^cdeX^	72.28 ^cY^	0.47	0.020	0.78 ^dY^	2.05 ^deX^	0.30	0.030	7.78 ^deY^	11.64 ^efX^	0.58	<0.001
Back	74.89 ^bcdX^	72.58 ^cY^	0.33	<0.001	2.84 ^cY^	4.76 ^bcX^	0.32	0.001	13.17 ^cY^	15.21 ^deX^	0.40	0.009
Breast interior	51.95 ^g^	51.59 ^f^	0.67	0.791	−0.94 ^eY^	0.80 ^efX^	0.20	<0.001	6.53 ^efY^	10.93 ^fX^	0.46	<0.001
Breast surface	54.55 ^g^	54.50 ^e^	0.46	0.959	−0.32 ^deY^	1.02 ^efX^	0.17	<0.001	7.39 ^deY^	10.58 ^fX^	0.45	<0.001
Drumette	72.71 ^deX^	71.31 ^cY^	0.31	0.021	3.46 ^bcY^	5.12 ^bX^	0.31	0.005	14.35 ^bcY^	17.75 ^cdX^	0.45	<0.001
Ham	66.05 ^f^	65.81 ^d^	0.48	0.805	−0.43 ^eY^	0.25 ^fX^	0.12	0.002	4.62 ^f^	5.47 ^g^	0.42	0.320
Hock	77.49 ^ab^	76.19 ^a^	0.35	0.057	−0.36 ^deY^	2.14 ^deX^	0.30	<0.001	16.39 ^abY^	21.52 ^bcX^	1.23	0.035
Neck	75.97 ^bcX^	73.49 ^bcY^	0.37	<0.001	3.19 ^bcY^	5.17 ^bX^	0.28	<0.001	14.98 ^bcY^	19.70 ^bcX^	0.52	<0.001
Saddle	73.02 ^de^	73.00 ^c^	0.34	0.975	4.31 ^ab^	5.08 ^b^	0.26	0.137	16.58 ^abY^	18.57 ^cdX^	0.44	0.020
Shank	78.99 ^aX^	75.84 ^abY^	0.32	<0.001	−0.27 ^deY^	3.32 ^cdX^	0.33	<0.001	18.70 ^aY^	31.59 ^aX^	1.23	<0.001
Thigh	72.93 ^de^	71.57 ^c^	0.37	0.064	−0.39 ^de^	0.15 ^f^	0.14	0.062	9.50 ^d^	10.86 ^f^	0.57	0.237
Vent	72.09 ^e^	71.93 ^c^	0.37	0.830	5.30 ^aY^	6.78 ^aX^	0.31	0.015	18.65 ^aY^	23.03 ^bX^	0.58	<0.001
SEM	0.63	0.59			0.17	0.19			0.39	0.56		
*P*	<0.001	<0.001			<0.001	<0.001			<0.001	<0.001		

^a, b, c, d, e, f, g^: mean values within feed with different letters differ significantly between carcass areas (*p* ≤ 0.05). ^X,Y^: mean values within location and variable with different letters differ significantly between diets (*p* ≤ 0.05). SEM: standard error of the mean.

**Table 4:** Chroma, Hue°, and ΔE of different carcass areas in broilers fed wheat- or corn-based diets.

	Chroma*		SEM	*p*-Value	Hue°		SEM	*p*-Value	ΔE
	WHEAT	CORN			WHEAT	CORN			
*n*	15	15			15	15			
Apterial latero-pectoral area	7.89 ^efY^	11.90 ^eX^	0.60	<0.001	84.91 ^cd^	80.81 ^cd^	1.48	0.169	4.60
Back	13.50 ^dY^	16.04 ^dX^	0.43	0.002	77.93 ^deX^	72.55 ^eY^	1.12	0.014	3.63
Breast interior	6.62 ^fgY^	10.98 ^eX^	0.46	<0.001	97.58 ^abX^	85.91 ^abcY^	1.33	<0.001	4.75
Breast surface	7.41 ^efY^	10.65 ^eX^	0.46	<0.001	93.05 ^bcX^	84.56 ^bcY^	1.06	<0.001	3.46
Drumette	14.80 ^cdY^	18.53 ^cdX^	0.49	<0.001	76.70 ^de^	73.92 ^e^	0.86	0.106	4.03
Ham	4.76 ^g^	5.50 ^f^	0.40	0.365	102.70 ^aX^	88.90 ^abY^	3.36	0.037	1.12
Hock	16.40 ^bcY^	21.67 ^bcX^	1.24	0.030	91.29 ^bcX^	84.14 ^bcY^	0.94	<0.001	5.92
Neck	15.34 ^cdY^	20.40 ^bcX^	0.55	<0.001	78.06 ^deX^	75.25 ^deY^	0.71	0.047	5.69
Saddle	17.16 ^abcY^	19.29 ^cdX^	0.46	0.018	75.64 ^de^	74.60 ^e^	0.65	0.429	2.13
Shank	18.71 ^abY^	31.77 ^aX^	1.24	<0.001	90.98 ^bcX^	84.06 ^bcY^	0.64	<0.001	13.75
Thigh	9.53 ^e^	10.90 ^e^	0.57	0.232	93.04 ^bc^	90.74 ^a^	0.89	0.200	2.00
Vent	19.45 ^aY^	24.02 ^bX^	0.61	<0.001	74.35 ^e^	73.64 ^e^	0.62	0.573	4.62
SEM	0.40	0.58			0.89	0.58			
*P*	<0.001	<0.001			<0.001	<0.001			

^a, b, c, d, e, f, g^: mean values within feed with different letters differ significantly between carcass areas (*p* ≤ 0.05). ^X,Y^: mean values within location with different letters differ significantly between diets (*p* ≤ 0.05). SEM: standard error of the mean.

## Data Availability

The original contributions presented in the study are included in the article, further inquiries can be directed to the corresponding author.
